# White matter microstructural changes in tuberous sclerosis: Evaluation by neurite orientation dispersion and density imaging (NODDI) and diffusion tensor images

**DOI:** 10.1038/s41598-019-57306-w

**Published:** 2020-01-16

**Authors:** Toshiaki Taoka, Noriko Aida, Yuta Fujii, Kazushi Ichikawa, Hisashi Kawai, Toshiki Nakane, Rintaro Ito, Shinji Naganawa

**Affiliations:** 1 Department of Innovative Biomedical Visualization, Graduate School of Medicine, Nagoya University, 65 Tsurumai-cho, Showa-ku, Nagoya, 466-8550 Aichi Japan; 2 Department of Radiology, Nagoya University, 65 Tsurumai-cho, Showa-ku, Nagoya, Aichi 466-8550 Japan; 3 Department of Radiology, Kanagawa Children’s Medical Center, 2-138-4 Mutsukawa, Minami-ku, Yokohama, 232-8555 Kanagawa Japan; 4 Department of Neurology, Kanagawa Children’s Medical Center, 2-138-4 Mutsukawa, Minami-ku, Yokohama, 232-8555 Kanagawa Japan

**Keywords:** Developmental disorders, Diagnostic markers, Paediatric neurological disorders

## Abstract

Neurite orientation dispersion and density imaging (NODDI) is a novel diffusion method for evaluating tissue microstructure, and may provide additional information over conventional diffusion tensor imaging (DTI). We evaluated NODDI and DTI parameters in cases of tuberous sclerosis (TS) to assess microstructural changes in the white matter. Eleven cases of tuberous sclerosis and eight age-matched controls underwent NODDI and DTI. We performed qualitative analysis and tract-based spatial statistics (TBSS) analysis of the NODDI parameters (Ficv: intracellular volume fraction, Fiso: isotropic fraction, ODI: orientation dispersion index) as well as DTI parameters (MD: mean diffusivity, FA: fractional anisotropy). We also performed a correlation analysis between clinical symptoms and parameters. The qualitative analysis indicated that the Ficv had a lower value in TS cases particularly in the tubers adjacent to the white matter. The TBSS analysis showed that the TS cases had decreased Ficv in a greater area compared to the other parameters including MD. In particular, the Ficv was decreased in deep white matter, such as the superior longitudinal fascicles (SLF). The application of NODDI to TS cases revealed tissue microstructural changes, and particularly the Ficv could detect more widespread abnormalities in white matter structure compared to DTI parameters.

## Introduction

Tuberous sclerosis (TS) is a multi-system genetic disease which causes hamartomatous lesions in multiple organs such as the brain, kidneys, heart, eyes, lungs, and skin. A mutation in either one of two (TSC1, TSC2) tumor suppressor genes may be responsible. The major pathological abnormalities in the central nervous system (CNS) are cortical tubers and subependymal nodules, which may arise from abnormalities of cortical proliferation, migration and organization^1^. The CNS pathology of TS includes linear abnormalities in the cerebral white matter, which are suggestive of myelination or migration disorders. These may include disorganized migration of neuronal cells in the developing cortex^2^. Although seizures and mental disorders are the major CNS symptoms in TS, the relationship between the observed pathology and symptoms is not known. According to previous reports, there is no consistent correlation between seizure symptoms and the number and location of the tubers. Neither a high tuber load nor tubers in specific locations are necessary or sufficient to predict seizures, cognitive impairment, or autism^3–5^. Therefore, it has been suggested that TS symptoms may arise from abnormal connections that are independent of tubers^6,7^. An imaging technique that can probe such changes in connectivity would help resolve the microstructural mechanisms of TS.

Diffusion tensor imaging (DTI) is a model for water diffusion and evaluate or visualize microstructural aspects of the brain particularly in white matter^8^. In tuberous sclerosis, DTI has been applied to evaluate white matter alterations. One report indicated that an abnormal mean diffusivity (MD) was not limited to perituberal white matter, but was also observed in perilesional normal appearing white matter^9^. However, other reports indicate no significant differences in either MD or fractional anisotropy (FA) in normal-appearing white matter of patients with TS compared to controls^10,11^. Aside from these examples, findings for DTI in TS remain unclear^12^.

DTI is based on an assumption of Gaussian distribution. With an appropriate b-value acquisition, the Gaussian assumption in DTI is presumed to be valid in clinical practice. However, it may not be appropriate to assess biological tissues in which water diffusion does not follow a Gaussian distribution. Techniques like diffusion kurtosis imaging (DKI) or diffusion spectrum imaging (DSI) can capture non-Gaussian properties, which provide more information about the underlying microstructure^13^. Neurite orientation dispersion and density imaging (NODDI) is an advanced diffusion imaging technique that provides detailed information of tissue microstructure in the brain^14–16^. NODDI is a multi-compartment biophysical tissue model and requires assumptions about neuronal tissues to provide a set of indices that are related to white matter microstructure. Like DSI, NODDI addresses the presence of multiple diffusion environments in each imaging unit, for which DTI and DKI do not. Both DTI and NODDI use diffusion MRI, which is inherently sensitive to tissue microstructure. The key advantage of NODDI is that it models the biophysical properties of the tissue and thus provides indices that are more directly related to the microstructure. This non-Gaussian diffusion model can quantitatively evaluate specific microstructural changes in terms of neurite density and the orientation distribution of neurites, which are axons and dendrites. The resultant parameters include maps of the intra-cellular volume fraction (Ficv) or neurite density, which is based on intracellular diffusion, the isotropic volume fraction (Fiso), which represents the free water compartment within the tissue, and the orientation dispersion index (ODI), which reflects the dispersion of neurites.

The purpose for the current study was to evaluate the Ficv, Fiso and ODI images generated by NODDI in tuberous sclerosis cases compared to the FA and MD images from conventional DTI in order to assess the microstructural changes of the white matter. The correlation between these parameters and clinical symptoms was also evaluated. As far as we know, this study is the first to apply NODDI techniques to TS cases in a clinical population.

## Results

### Qualitative analysis of the white matter containing cortical tubers in comparison to controls

**Figure 1:**
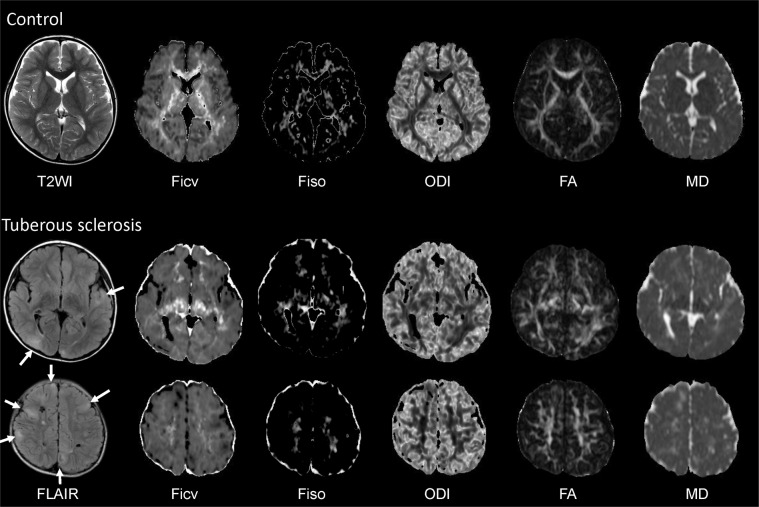
NODDI and diffusion tensor images of a control and a tuberous sclerosis (TS) case. Control (Upper row): 8-year-old female. TS case (Middle and lower row): 14-year-old female (Middle row: images at the level of the thalamus, Lower row: images at the level of the semioval center) For the control, the T2WI, and the Ficv, Fiso, ODI, FA and MD maps are shown respectively. The Ficv had higher values in the white matter, particularly in the projection fibers of the deep white matter. The intensity in the deep white matter had higher values of the Fiso as well. The ODI image indicated that the dispersion of the fiber orientation was smaller in the white matter especially in the deep white matter. The FA maps of the controls had higher values in the white matter, and the MD map indicated lower values in the white matter. For the TS case, the FLAIR images show cortical tubers (arrows) and white matter lesions as increased signal intensity. The Ficv map shows a lower intensity compared to the control both in the deep white matter (projection area) and the subcortical white matter (association area). On the Fiso map, the deep white matter has rather higher intensity, while the subcortical white matter is very low. On the ODI and FA maps, the control and TS case look very similar qualitatively. On the MD map, the cortical tubers and white matter lesions have a high signal intensity.

**Figure 2:**
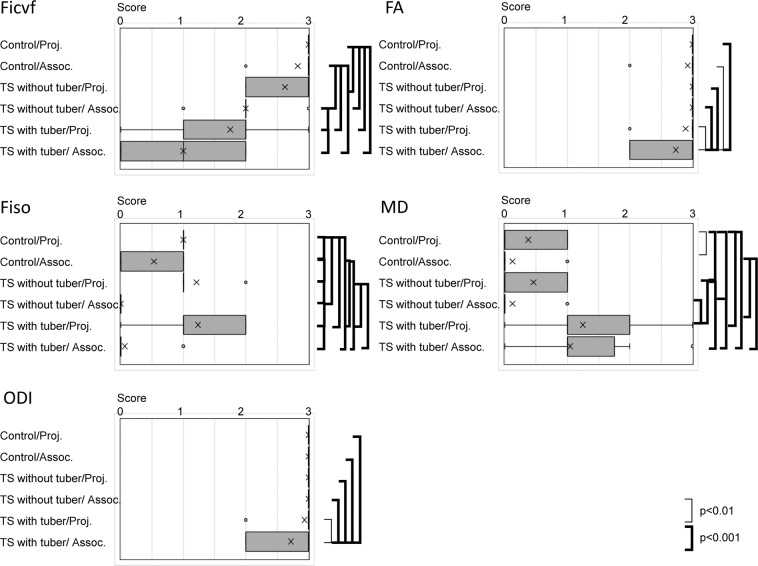
Qualitative evaluation of NODDI and diffusion tensor images from controls and TS cases. The scores of the qualitative analysis of the NODDI and diffusion tensor imaging parameters (Ficv, Fiso, ODI, FA and MD) are shown in box and whisker plot. Projection fiber (Proj.) and association fiber (Assoc.) regions in the frontal, parietal, occipital and temporal lobes were evaluated. For the images in which the normal white matter had a higher signal intensity than the gray matter (Ficv, Fiso and FA), the grading was as follows 3: Higher than gray matter all over the area, 2: Higher than gray matter in more than half of the area, 1: Higher than gray matter in less than half of the area and 0: Same as or lower than gray matter. For the images in which the normal white matter had a lower signal intensity (ODI and MD), the intensity of the white matter was scored as 3: Lower than gray matter all over the area, 2: Lower than gray matter in more than half of the area,, 1: Lower than gray matter in less than half of the area and 0: Same as or higher than gray matter. Statistical differences were evaluated by a Tukey-Kramer Test.

Representative NODDI and DTI images are provided in Fig. 1. The result of the qualitative analysis is indicated in Fig. 2 as box and whisker plots with the statistical differences assessed by a Tukey-Kramer Test. The Ficv in TS cases had decreased scores, particularly in the association fibers containing cortical tubers. Even within the white matter without tubers, the qualitative estimation of the Ficv resulted in decreased scores compared to controls. On the MD maps, the TS cases had increased scores compared to controls, particularly in regions adjacent to the cortical tubers. The qualitative evaluation of the ODI and FA maps indicated only small differences between the TS cases and controls. However, in the association fiber areas with cortical tubers there were decreased scores compared to controls. The Fiso had lower scores in the TS cases in the association fiber regions, and the projection fibers had higher scores.

### Quantitative analysis of the NODDI and diffusion tensor parameters

**Figure 3:**
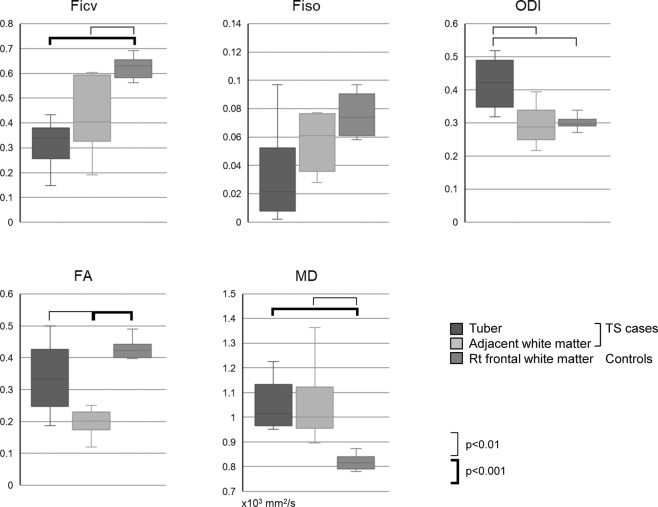
Quantitative evaluation of NODDI and diffusion tensor images from controls and TS cases. The ROIs were placed on the largest tuber in the TS cases, and ROIs were also placed in the white matter area adjacent to the largest tuber in the TS cases. For the controls, ROIs were placed in the white matter of the right frontal lobe. Box and whisker plots are show for the values of the NODDI parameters and diffusion tensor parameters including Ficv, Fiso, ODI, FA and MD. The ROIs (150 to 230 mm^2^ area) were placed on the largest tuber in the TS cases, and ROIs (250 to 300 mm^2^ area) were also placed in the white matter area adjacent to the largest tuber in the TS cases. For the controls, ROIs (250 to 300 mm^2^ area) were placed in the white matter of the right frontal lobe and the above-mentioned values were measured.

Region of interest (ROI) based analysis was made for the NODDI parameters and diffusion tensor parameters including Ficv, Fiso, ODI, FA and MD. The ROIs (150 to 230 mm^2^ area) were placed on the largest tuber in the TS cases, and ROIs (250 to 300 mm^2^ area) were also placed in the white matter area adjacent to the largest tuber in the TS cases. For the controls, ROIs (250 to 300 mm^2^ area) were placed in the white matter of the right frontal lobe and the above-mentioned values were measured. ANOVA indicated the statistically significant differences between the area shown in the Fig. 3 including Ficv and MD between tubers in the TS cases and white matter in the controls.

**Figure 4:**
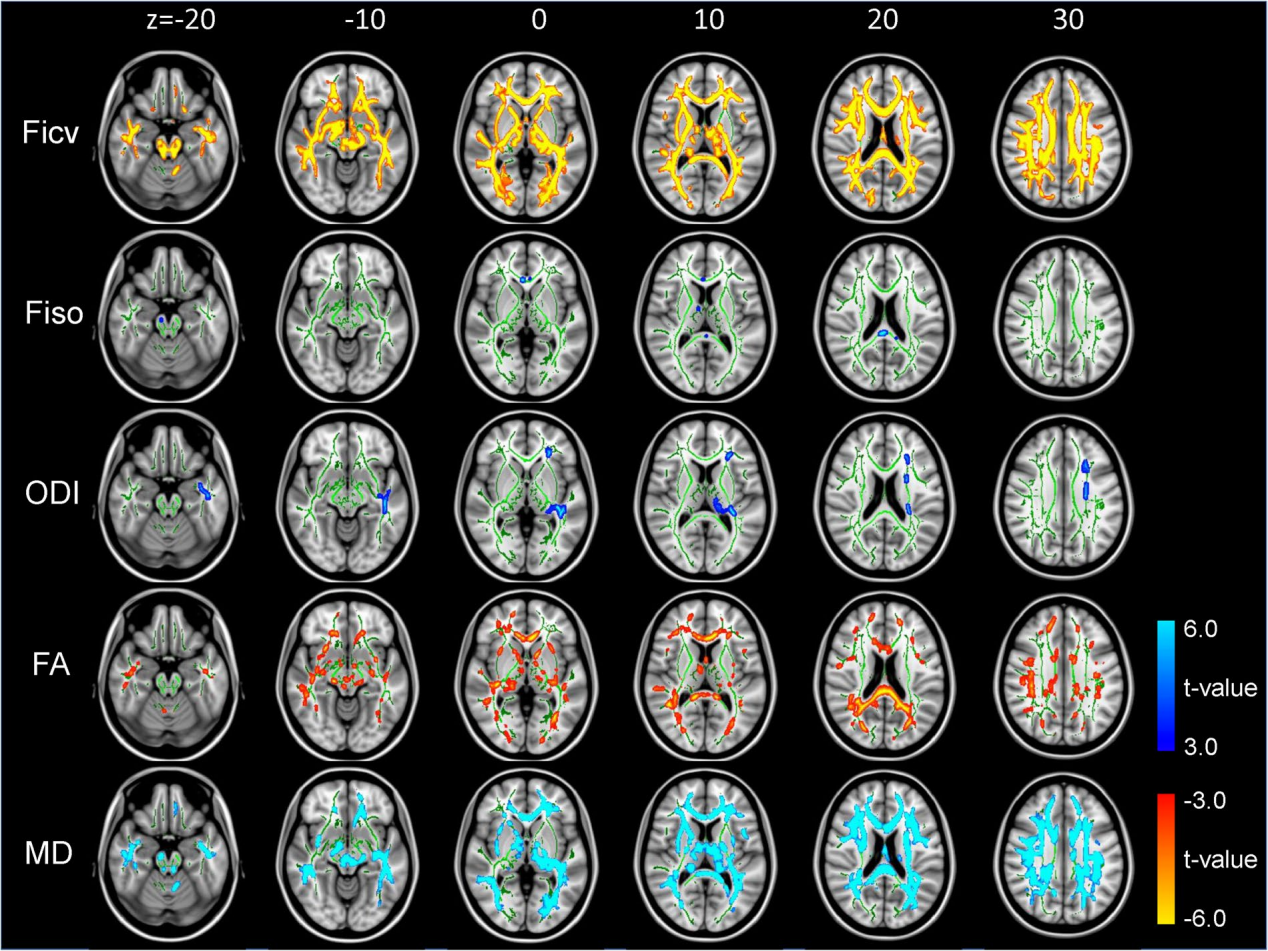
Tract-based spatial statistics (TBSS) analysis of the NODDI and diffusion tensor parameters. Ficv, Fiso, ODI, FA and MD were tested for differences between controls and TS cases. The thresholded t-stat images (t > 3.0 for Fiso, ODI and MD, t < −3.0 for Ficv and FA) were generated and evaluated for the differences between TS cases and controls. (For full t-stat maps, refer the supplementary information). The Ficv maps had significant decreases in TS cases in widespread regions including the short association fibers of the cerebrum, projection fibers and superior longitudinal fascicles (SLF) in the corona radiate, external capsule, and the corpus callosum including the genu and splenium.

Tract-based spatial statistics (TBSS) analysis was also made. The FA skeleton of the white matter fasciculus was compared between controls and TS cases and t-stat images (p-value images) for the differences were generated (Fig. 4). On the t-stat images for the Ficv, wide spread brain regions indicated reduced Ficv in the TS cases, including the short association fibers of the frontal, parietal, temporal and occipital lobes. In addition, a region including the projection fibers and the superior longitudinal fascicles (SLF) in the corona radiate also had decreased Ficv values. Decreased Ficv values were also seen in the external capsule, and the corpus callosum including the genu and splenium. On the t-stat images for the Fiso, a small region within the association fibers of the frontal and parietal lobes had decreased values. On the t-stat images for the ODI, a small region within the association fibers of the frontal and parietal lobes and a small region within the SLF had increased ODI values. On the t-stat images for FA, rather small regions within the association fibers of the frontal, parietal, temporal and occipital lobes had decreased FA values. Decreased FA values were also seen in the external capsule, and corpus callosum including the genu and splenium. On the t-stat images for the MD, a small region within the association fibers of the frontal and parietal lobes and a small area within SLF were increased.

The overall brain volumes calculated from brain mask images which were used in the process of NODDI images generation were 1242.0 cm^3^ in the mean (standard deviation: 137.0 cm^3^) for the TS cases and 1167.5 cm^3^ in the mean (standard deviation: 128.3 cm^3^) for the controls. There was no statistically significant difference between the TS cases and the controls by Student’s t-test.

### Correlations of clinical symptoms with NODDI and DTI parameters

The clinical symptoms of the TS cases including mental retardation, myoclonic seizures and intractable epilepsy are shown in Table 1. Table 2 presents the Spearman’s rank correlation coefficients (r_s_) and the p-values for the correlations between clinical symptoms and the parameters, which included the Ficv, Fiso, ODI, FA and MD from regions of interest (ROIs) placed in the superior longitudinal fasiculus (SLF), internal capsule, external capsule and the corpus callosum on the individual images. None of the regions had statistically significant correlations (p < 0.05).

**Table 1:** Clinical symptoms of tuberous sclerosis cases.

Age	Sex	Mental disorder	Myoclonic seizure	Intractable epilepsy
5	F	Moderate	−	−
5	M	None	−	+
6	F	Mild	−	−
7	M	Moderate	+	−
8	F	Severe	+	+
8	F	None	−	−
12	M	Mild	−	−
13	M	Mild	+	−
14	F	Severe	+	+
14	M	Severe	+	+

**Table 2:** Sperman’s rank correlation coefficient (r_s_) and the p-value between clinical symptoms and parameters from NODDI and DTI.

		Mental reterdation		Myoclonic seizures		Intractable epilepsy	
		r_s_	*p*	r_s_	*p*	r_s_	*p*
Ficv	SLF	−0.41	0.22	−0.10	0.75	0.14	0.65
	Internal capsule	−0.31	0.35	−0.10	0.75	−0.07	0.83
	External capsule	0.09	0.78	−0.10	0.75	0.14	0.67
	Corpus callosum	−0.03	0.93	−0.03	0.92	−0.50	0.14
Fiso	SLF	−0.34	0.30	−0.17	0.60	0.14	0.67
	Internal capsule	0.27	0.42	0.29	0.14	0.23	0.20
	External capsule	−0.09	0.78	−0.17	0.60	−0.28	0.39
	Corpus callosum	−0.22	0.51	−0.31	0.35	0.21	0.52
ODI	SLF	0.59	0.07	0.38	0.25	0.36	0.29
	Internal capsule	0.28	0.40	0.17	0.60	0.50	0.14
	External capsule	0.41	0.22	0.10	0.75	0.14	0.67
	Corpus callosum	0.28	0.40	0.52	0.12	0.28	0.39
FA	SLF	0.06	0.85	0.28	0.25	−0.11	0.75
	Internal capsule	0.02	0.96	0.14	0.68	0.29	0.39
	External capsule	−0.06	0.85	−0.31	0.35	0.07	0.83
	Corpus callosum	−0.09	0.78	−0.10	0.75	0.14	0.67
MD	SLF	0.25	0.45	0.17	0.60	0.21	0.52
	Internal capsule	−0.03	0.93	0.03	0.92	0.28	0.39
	External capsule	0.19	0.57	0.10	0.75	0.36	0.29
	Corpus callosum	0.44	0.19	0.10	0.75	0.36	0.29

SLF: superior longitudinal fascicles.

## Discussion

The NODDI parameters reflect the white matter microstructure^14–16^. In the current study, there was a trend to indicate that the Ficv, which is defined as the intra-neurite tissue volume fraction and represents the density of neurites, was reduced in the white matter of TS cases. The qualitative analysis indicated that the white matter of TS cases had decreased Ficv, particularly in regions with apparent cortical tubers. The TBSS analysis also showed significant decreases of Ficv in widespread regions within the brain, and the Ficv delineated the white matter changes more sensitively compared to the other parameters (Fiso, ODI, FA and MD) investigated in this study. The MD, which describes general diffusion is typically representative of increased water content within the tissue. Qualitative analysis of the MD maps indicated increased MD in TS cases, particularly in regions adjacent to cortical tubers. Quantitative analysis for the ROIs in the largest tuber in the TS cases and the white matter in the controls indicated the decreased Ficv and increased MD in the tubers. While, a global comparison by TBSS analysis indicated that the regions with increased MD were limited to a few areas including the corpus callosum. Although cortical tubers are reported to have high MD values^17^, the white matter changes in TS are barely detectable by MD, which is derived from single-shell diffusion tensor data. The Fiso represents the ratio of free water in tissue, and in this study, we found a slightly higher value in the TS cases using TBSS analysis. The Fiso in the SLF was slightly correlated to the severity of mental retardation. It is interesting that the qualitative analysis indicated the Fiso was decreased in the association fibers and increased in the projection fibers. ODI quantifies the orientation dispersion of the fibres, which may arise from dispersion or bending/fanning of a single fibre population or crossing of multiple populations. In the current study, ODI was very slightly increased in the TS cases. With both the qualitative study and the TBSS analysis, the ODI maps as well as FA maps indicated little difference between controls and TS cases. This observation may indicate that the underlying white matter organization, which is assessed by the ODI or FA, is preserved in TS. Alternatively, the neurite density, which is indicated by the Ficv, is decreased in the white matter of the TS cases.

Although conventional MRI has been used for the diagnosis of TS brain lesions, it is known that tuber-like pathology distributes diffusely in the white matter below the detection level of conventional MRI. Thus, normal appearing white matter in TS may have an abnormal microstructure^9,12,18^. Regarding alterations of normal appearing white matter in TS, there is a report of diffusion weighted imaging showing significant MD increases in the supratentorial normal appearing white matter with TS^9^. Another report of 7 TS cases indicated that the MD values were higher and the FA values were lower in white matter lesions and perilesional white matter^10^. However, no significant differences in either MD or FA values in normal appearing white matter were found. Alternatively, a diffusion tensor study of 23 TS cases indicated that the MD values of normal appearing white matter were increased in TS patients, suggesting that myelination may be delayed/impaired in TS patients. This finding may explain the global neurocognitive deficits in these subjects^19^. In the white matter of TS patients, abnormal cells including atypical astrocytes may be scattered widely throughout the deep white matter. A pathology study also indicated reduced myelin density, activated microglial cells and disruption of blood-brain barrier permeability in TS^20^. The finding of reduced Ficv in the white matter may reflect decreased neurite density or axonal loss in association with the above-mentioned microstructural abnormalities. Increased MD in TS has been reported by many previous studies in agreement with our finding^9,17,19,21^. Our results indicate that the MD in the corpus callosum had a weak positive correlation with the severity of mental retardation. The FA and ODI values were not significantly different between TS cases and controls. This finding may indicate that the underlying white matter fiber structure is preserved in TS despite the pathological changes, which included the appearance of abnormal cells and decreased neurite density. The qualitative findings of our study showed that the Fiso was increased in the projection fibers, which contain periventricular regions. This finding may be due to cyst-like or microcystic abnormalities, which can be found in the periventricular area in TS^22^. The SLF is a long association fiber tract, which connects the anterior and posterior cortices in the fronto-parietal regions and is related to higher cortical functions. Alterations of white matter tissue in this region may lead to impaired mental function. The current study indicated a slight correlation between the degree of mental retardation and decreased Ficv, decreased Fiso, as well as increased ODI. These observations may indicate that the NODDI parameters delineate the microstructural alterations in TS, which lead to functional deterioration.

This study has some limitations. First, the numbers of the TS cases and controls were small. This point may have influenced the statistical power and led to the negative results in the current study. In addition, genetic information could not be obtained and we could not analyze the associations between image findings and genetic abnormalities.

The slice thicknesses of the diffusion images are 3 mm in the current study. Thus the voxel of the diffusion images were anisotropic and this may alter the parameter values. However, the subjects of this study were from a pediatric population and we could not scan over long periods. Resolution or partial volume of the images are reported to influence the skeleton shape in TBSS analysis^23^. In order to validate the influence of rather larger acquisition voxels in the current study, we calculated the overall brain volumes and compared between TS cases and controls. Since there was no statistically significant difference in the overall brain volume, the influence of the lower resolution in the current study might be small. The qualitative evaluation of this study had only 3 steps, which was another limitation. The main purpose of the qualitative assessment was to illustrate the difference between regions with and without tubers, so we considered that we did not need to make a detailed assessment of the parameters. We made the qualitative evaluation by the frontal, parietal, occipital and temporal lobes, so the size of the areas for evaluation were not the same. In the current study, NODDI images were generated from the image of b = 0, 1000 and 2000 s/mm^2^, and DTI images including FA and ADC (MD) images were generated from the image of b = 0 and 1000 s/mm^2^ from the same datasets. Thus, DTI and NODDI fitting were performed with different number of volumes and different softwares. These factors could contribute to the discrepancy of DTI vs NODDI results.

In conclusion, NODDI imaging of TS cases seemed to indicate tissue microstructure changes. The Ficv indicated that the neurite density is decreased in TS in widespread regions including deep white matter tracts such as the SLF. A high MD value may correspond to histological abnormalities including abnormal neuronal cells, but MD did not seem to delineate white matter changes. The ODI and FA values did not show large differences, which may indicate preservation of the underlying white matter framework.

## Methods

### Subjects

This study was approved by the institutional review boards of the participating institutions (Nagoya University Hospital Institutional review board, Institutional review board of Kanagawa Children’s Medical Center). Written informed consent for retrospective image processing was waived by the institutions based on obtaining a comprehensive agreement from patients on admission to use anonymized medical information. Written informed consent was obtained from the normal volunteers or their parents.

Eleven cases (Male: 6, Female: 5) with tuberous sclerosis which met the clinical diagnostic criteria of the 2012 International Tuberous Sclerosis Complex Diagnostic Criteria^24^ as “definite diagnosis” or “possible diagnoses” were enrolled. The image acquisitions were undergone from March 2013 to June 2016. All the newly admitted TS cases of the period were examined. The age ranged from 5 to 14 years old. The clinical symptoms including mental disorders, myoclonic seizures and intractable epilepsy are provided in Table 1. We also enrolled eight age-matched (range 6 to 16 years old) controls who underwent identical imaging.

### Image acquisition

Imaging was performed on a 3.0 T clinical scanner (Magnetom Verio, Siemens AG, Erlangen, Germany). The image acquisition included T2-weighted images (T2WI, turbo spin echo, TR = 4500 ms, TE = 90 ms, FA = 145 degrees, FOV = 230 × 230 mm, matrix = 512 × 512, slice thickness = 4.5 mm), FLAIR images (turbo spin echo, TR = 10000 ms, TE = 90 ms, TI = 2200 ms, FOV = 230 × 230 mm, matrix = 512 × 512, slice thickness = 4.5 mm) and a diffusion image for the NODDI (echo planar, TR = 6600 ms, TE = 89 ms, b = 0,1000,2000 s/mm^2^, motion probing gradient = 30 directions, FOV = 230 mm, matrix = 94 × 94, slice thickness = 3 mm, acquisition time = 7.02 min).

### Image processing and analysis

We used MATLAB R2014a with the optimization toolbox on a workstation, and NODDI images including the Ficv, ODI and Fiso maps were generated by using the NODDI Matlab toolbox (http://mig.cs.ucl.ac.uk/index.php?n=Download.NODDI) developed by University College London. As pre-processing for NODDI, Eddy current correction and brain masking were applied. The NODDI imaging generates maps of Ficv representing neurite density, ODI representing the orientation dispersion of the fibers and Fiso representing the fraction of free water. The MD and FA images were generated by the console software of the MR scanner (VB15A) by using images of b = 0 and b = 1000 s/mm^2^ from the same datasets which were acquired for NODDI generation.

We evaluated the T2 weighted and FLAIR images for the existence of cortical tubers in the TS cases by each cerebral lobe using qualitative analysis. On the NODDI and diffusion tensor images, we performed the following: (1) a qualitative scoring analysis of the images comparing the cerebral lobes with and without apparent cortical tubers, (2) Tract-based spatial statistics (TBSS), which is a quantitative voxel-wise analysis of the images to evaluate the distribution of the NODDI and diffusion tensor values, and (3) a statistical analysis between the quantitative value on the images and the clinical symptoms as follows.

(1) Qualitative analysis of the white matter in TS and controls by scores. For TS, the score was calculated in relation to the existence of cortical tubers. On the Ficv, Fiso and FA images, the intensity of the white matter was scored as 3: Higher than gray matter all over the area, 2: Higher than gray matter in more than half of the area, 1: Higher than gray matter in less than half of the area and 0: Same as or lower than gray matter. For the ODI and MD images, the intensity of the white matter was scored as 3: Lower than gray matter all over the area, 2: Lower than gray matter in more than half of the area,, 1: Lower than gray matter in less than half of the area and 0: Same as or higher than gray matter. The locations used for the analysis were the projection and association fibers of the frontal, parietal, occipital and temporal lobes. Areas for the projection and the association fibers are determined by color display of the diffusion tensor images generated by the console software of the MR scanner, and the areas of cerebral lobes were recognized by the positions of sulci. We classified the areas by the presence of cortical tubers on T2WI and FLAIR, and the scores of the areas with and without tubers were compared by box and whisker plot and the statistical differences were evaluated by a Tukey-Kramer Test.

(2) Quantitative analysis of the NODDI and diffusion tensor parameters. Region of interest (ROI) based analysis was made for the NODDI parameters and diffusion tensor parameters including Ficv, Fiso, ODI, FA and MD. In the TS cases, the ROIs were placed on the largest tuber and in the white matter area adjacent to the largest tuber. For the controls, ROIs were placed in the white matter of the right frontal lobe. Analysis of Variance (ANOVA) was made for the statistical difference between three areas.

The NODDI and diffusion tensor images including the Ficv, Fiso, ODI, FA and MD images were evaluated by TBSS using Functional Magnetic Resonance Imaging of the Brain (FMRIB) Software Library (FSL)^25^. The FA maps from all subjects were aligned to an FA standard template by a nonlinear coregistration. The aligned FA maps were then averaged to produce a group mean image, and FA skeletons were generated highlighting the tracts common to the entire group. For each subject, an FA threshold of 0.2 was used before projecting the aligned FA map onto this skeleton. In addition to the FA map, the Ficv, Fiso, ODI and MD maps were generated using the same steps. Using an FSL permutation test of 500 permutations, the Ficv, Fiso, ODI, FA and MD maps were tested for differences between controls and TS cases. The unthresholded t-stat images (p-value images) were generated and evaluated for differences between TS cases and controls. In order to validate the influence of rather larger acquisition voxels in the current study^23^, we calculated the overall brain volumes from brain mask images generated from b = 0 mm/s^2^ images which were used in the process of NODDI images generation, and compared the volumes between TS cases and controls by Student’s t-test.

(3) Correlations between clinical symptom and image parameters. Clinical symptoms were evaluated including mental retardation, myoclonic seizures and intractable epilepsy (Table 1). The severity of mental retardation was classified into none (IQ higher than 70), mild (IQ of 50 to 70), intermediate (IQ of 35 to 50) and severe (IQ lower than 35). Myoclonic seizures and intractable epilepsy were classified into absence or existence. Clinical symptoms were correlated to the parameters including the Ficv, Fiso, ODI, FA and MD using ROIs placed in the superior longitudinal fascicles (SLF), internal capsule, external capsule and the corpus callosum on individual images, which were evaluated by a Spearman’s rank correlation test and calculated the Spearman rank coefficients (r_s_) and p-values^26^.

## Supplementary information

Supplement Figure.

## Data Availability

The datasets generated during and/or analysed during the current study are available from the corresponding author on reasonable request.
