# Supplementary material for: White matter microstructural changes in tuberous sclerosis: Evaluation by neurite orientation dispersion and density imaging (NODDI) and diffusion tensor images
Source: Sci Rep. 2020 Jan 16;10:436. doi: 10.1038/s41598-019-57306-w (PMC6965630; doi:10.1038/s41598-019-57306-w)

White matter microstructural changes in tuberous sclerosis: Evaluation by neurite orientation dispersion and density imaging (NODDI) and diffusion tensor images.

Toshiaki Taoka MD PhD[1], Noriko Aida MD PhD[2], Yuta Fujii MD[2],

Kazushi Ichikawa MD PhD[3], Hisashi Kawai MD PhD[1], Toshiki Nakane MD PhD[1],

Rintaro Ito MD PhD[1], Shinji Naganawa MD PhD[1]

1. Department of Radiology, Nagoya University, Nagoya, Japan

65 Tsurumai-cho, Showa-ku, Nagoya, Aichi 466-8550, Japan

2. Department of Radiology, Kanagawa Children's Medical Center

2-138-4 Mutsukawa, Minami-ku, Yokohama, 232-8555, Japan.

3. Department of Neurology, Kanagawa Children's Medical Center

2-138-4 Mutsukawa, Minami-ku, Yokohama, 232-8555, Japan.

Corresponding Author

Toshiaki Taoka, M.D. Ph.D.

Department of Radiology, Nagoya University Hospital

65 Tsurumai-cho, Showa-ku, Nagoya, Aichi 466-8550, Japan

Fax: +81-52-744-2335

Tel: +81-52-744-2327

E-mail: ttaoka@med.nagoya-u.ac.jp

Supplement Figure.

Full t-stat maps by tract-based spatial statistics (TBSS) analysis of the NODDI and diffusion tensor parameters

Ficv, Fiso, ODI, FA and MD were tested for differences between controls and TS cases. Full t-stat maps are shown. The Ficv maps had significant decreases in TS cases in widespread regions including the short association fibers of the cerebrum, projection fibers and superior longitudinal fascicles (SLF) in the corona radiate, external capsule, and the corpus callosum including the genu and splenium.

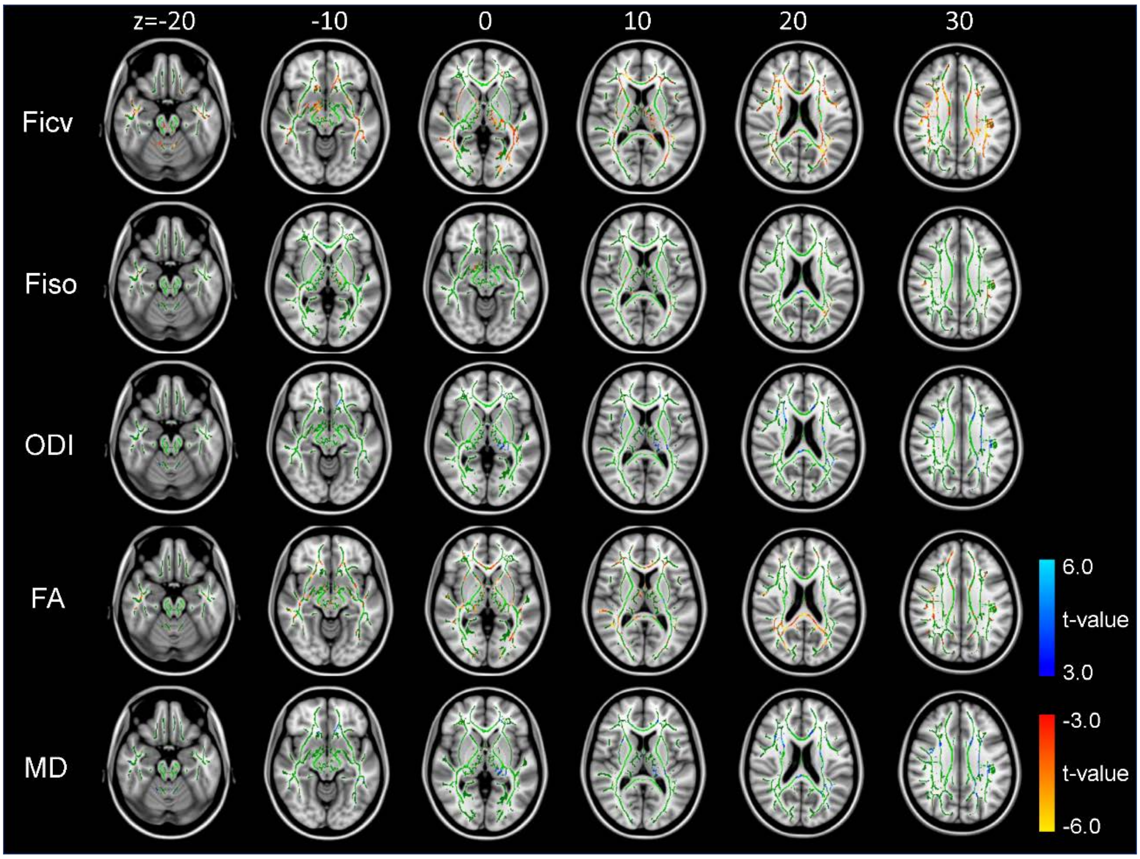

Supplement: Supplementary file 1 — Supplement Figure. [file 41598_2019_57306_MOESM1_ESM.pdf]
